# Uncommon Presentation of Gastric Duplication Cyst with Left-Sided Portal Hypertension: A Case Report and Literature Review

**DOI:** 10.3390/diagnostics14070675

**Published:** 2024-03-22

**Authors:** Adrian Boicean, Diana Prisca, Dan Georgian Bratu, Ciprian Ionut Bacila, Ciprian Tanasescu, Radu Chicea, Sorin Radu Fleaca, Sabrina Andreea Birsan, Cristian Ichim, Calin Ilie Mohor, Mihai Dan Roman, Adrian Nicolae Cristian, Samuel Bogdan Todor, Cosmin Ioan Mohor, Andrei Moisin, Adrian Hasegan

**Affiliations:** 1 Faculty of Medicine, Lucian Blaga University of Sibiu, 550169 Sibiu, Romania; adrian.boicean@ulbsibiu.ro (A.B.); ciprian.bacila@ulbsibiu.ro (C.I.B.); ciprian.tanasescu@ulbsibiu.ro (C.T.); radu.chicea@ulbsibiu.ro (R.C.); radu.fleaca@ulbsibiu.ro (S.R.F.); sabrina.marinca@yahoo.com (S.A.B.); cristian.ichim@ulbsibiu.ro (C.I.); calin.mohor@ulbsibiu.ro (C.I.M.); mihai.roman@ulbsibiu.ro (M.D.R.); adrian.cristian@ulbsibiu.ro (A.N.C.); samuelbogdant@gmail.com (S.B.T.); cosmin.mohor@ulbsibiu.ro (C.I.M.); andreicatalin.moisin@ulbsibiu.ro (A.M.); adrian.hasegan@ulbsibiu.ro (A.H.); 2 County Clinical Emergency Hospital of Sibiu, 550245 Sibiu, Romania; olariudiana@yahoo.com

**Keywords:** gastric duplication cyst, echo-endoscopy, left-sided portal hypertension

## Abstract

Gastric duplication cysts (GDCs) in adults are exceedingly rare, with only a few documented cases in medical literature. The spectrum of clinical presentations varies widely, ranging from asymptomatic to severe symptoms such as hematemesis, vomiting or abdominal pain. Among the less common complications associated with GDCs, segmental portal hypertension is a notable rarity. We present a compelling case report of a patient exhibiting signs of segmental portal hypertension, where ultrasound and echo-endoscopy revealed a sizable gastric duplication cyst as the underlying etiology. Recognizing the scarcity of literature on GDCs in adult patients, we conducted a thorough review to underscore the diagnostic significance of ultrasonography and endoscopic ultrasound (EUS) in accurately identifying these congenital anomalies. This case report and comprehensive literature review emphasize the pivotal role of EUS and abdominal ultrasound in achieving an accurate diagnosis of GDCs. By shedding light on the diagnostic and therapeutic intricacies, we aim to raise awareness among clinicians regarding this rare pathology and the importance of multimodal imaging approaches for optimal patient management.

## 1. Introduction

Gastrointestinal duplication cysts (GDCs) are congenital malformations usually diagnosed in children or young people. GDCs have a low incidence in adult patients, and they may be very easily misdiagnosed. Duplication cysts of the stomach account for as much as 8% of all alimentary tract duplications. At the moment, in the literature, there are two types of gastric duplication cysts: one type lacks communication with the gastric cavity or the tubular digestive tract, while the other type does communicate with the gastric lumen. When symptomatic, this type of pathology can cause a wider range of symptoms, such as persistent abdominal pain, nausea and vomiting or even life-threatening hematemesis [1,2,3,4,5].

Cases of GDCs complicated with portal hypertension are particularly rare, and their description in the literature is brief. The majority of patients are asymptomatic, possibly with alterations in liver function tests [6].

From a histological perspective, the embryonic origin of gut duplication cysts can be esophageal or bronchogenic. Such cysts appear to occur during gestation weeks 4–8, but studies have not definitively established the exact origin or malformation theory. Some embryonic defects that may explain duplication also include embryologic enteric diverticula and incomplete notochordal development [7,8].

In asymptomatic cases, GDCs may be discovered in screening endoscopy, and the aspect of gastric duplication cysts may be described as submucosal masses compressing the gastric cavity, but with a normal aspect of the gastric mucosa, without signs of infiltration. Endoscopically, this type of gastric malformation may be difficult to distinguish from other submucosal lesions, such as a leiomyoma or a gastrointestinal stromal tumor (GIST), due to the normal aspect of the overlying mucosa and the compressive pattern, which are representative of all these pathologies [9,10]. The current advances in imaging enable an improved ability to make the correct diagnosis and ensure better case management. One of the most advanced methods remains echo-endoscopy, an interventional echography, which can accurately diagnose duplication cysts by describing the mass characteristics, invasion into other organs, presence of adenopathy or vascular invasion [11,12,13].

Due to the frequently common symptoms with other pathologies and the imaging peculiarities of GDCs, diagnosing them is often challenging and can be mistaken. Thus, only 35% of patients had a preoperative diagnosis of GDC, confirming the significance and the niche in which this pathology exists [1]. GDCs can lead to obstruction, torsion, hemorrhage or rare cases of left-sided portal hypertension or malignancy, which have a poor prognosis, so a proper diagnostic is important [14,15]. When striving for an accurate diagnosis, other pathologies must also be taken into consideration, with malignant pancreatic cystic tumors (such as pancreatic mucinous cystadenoma) being mandatory in the differential diagnosis [16]. Pancreatic cystic tumors render different clinical symptoms, as well as other endoscopic ultrasound (EUS) features. Concrete, malignant pancreatic masses show solid and/or cystic lesions with anarchical vascularization and high concentrations of CA 19-9 [17].

The following case presents a rare occurrence of GDC, featuring an uncommon complication involving extrinsic compression on the splenic vein. This compression leads to left-sided portal hypertension, resulting in the formation of gastric varices through collateral vessels in the upper stomach. Additionally, due to the redirection of splenic blood flow through the coronary vein, esophageal varices have developed. This study also aims to highlight the clinical advantages of ultrasound and echo-endoscopy in accurately diagnosing duplication cysts and their potential complications, while also discussing optimal treatment options.

## 2. Case Report

A 68-year-old male presented to the gastroenterology department with complaints of abdominal pain, vomiting and weight loss with an onset about 6 months previously. The pain was described as occurring periodically, as having moderate severity, located in the epigastric region and extending towards the mesogastric area, with no relief from painkillers, analgesics or proton pump inhibitors. The patient’s medical background included hypertension but no alcohol consumption. Physical examination revealed abdominal tenderness without organomegaly, normal bowel sounds and a normal rectal digital examination. He had no history of surgical procedures or travel beyond Europe.

**Figure 1:**
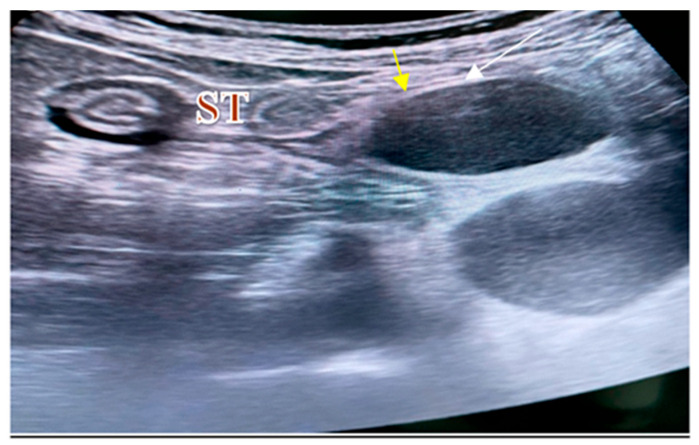
Ultrasound view of a GDC: the inner hyperechoic epithelial lining consists of the alimentary tract mucosa (white arrow) and the exterior hypoechoic layer of smooth muscle (yellow arrow), closely affixed to the gastrointestinal tract by sharing a common wall; ST: stomach. (Personal collection).

An abdominal ultrasound showed a normal liver surface, with no increased echogenicity or uniformity in parenchyma. Transient elastography, used to assess fibrosis, indicated stage F0. It should be noted that the ultrasonography established a 15 mm diameter portal vein in gentle breathing and a *double-wall* sign described as a hyperechoic mucosal band in a hypoechoic image representing the muscular layer (Figure 1). A gastric duplication cyst was suspected, considering the ultrasonography characteristics.

**Figure 2:**
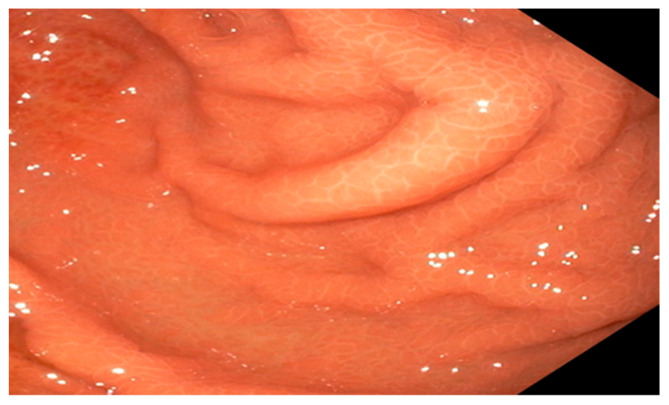
Endoscopic view of portal hypertensive gastropathy (Personal collection).

**Figure 3:**
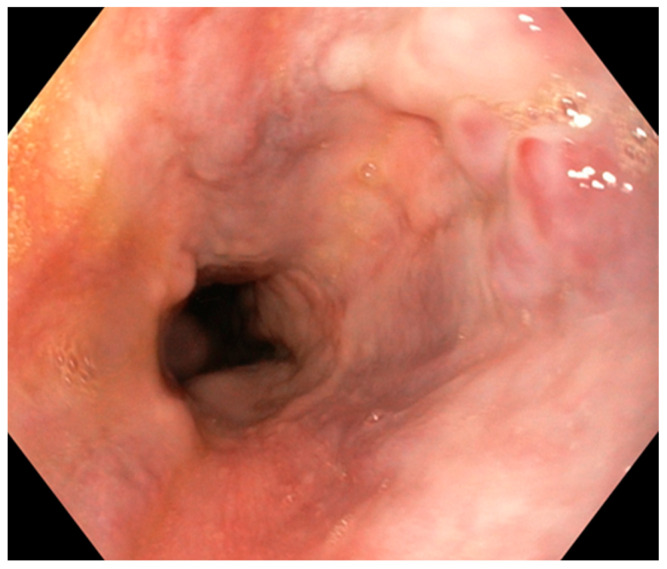
Endoscopic view of median esophageal varices (Personal collection).

**Figure 4:**
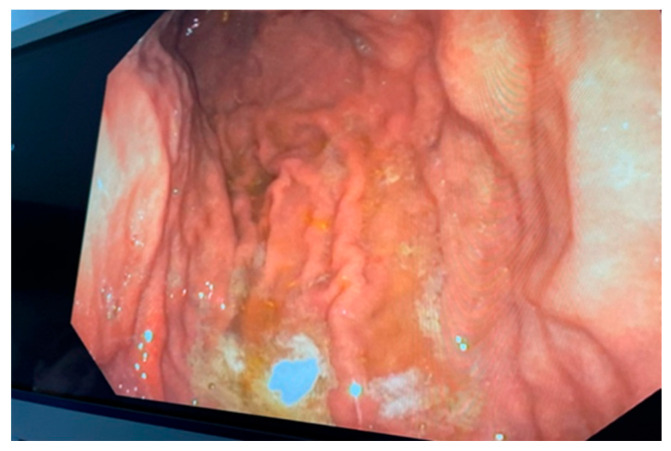
Endoscopic view of the stomach—a compressing mass on the greater curvature with a normal surface mucosa can be seen (Personal collection).

Upon performing a gastroscopy, some particularities raised a red flag for the clinician: grade 1/2 esophageal varices were observed in the upper third of the esophagus, as well as a compressing mass on the greater curvature and the gastric mucosa revealed portal gastropathy, without any mucosal infiltration or other lesions (Figure 2, Figure 3 and Figure 4). Due to the existence of grade 2 esophageal varices, propranolol was initiated at a dose of 2 × 20 mg/day.

Viral markers for chronic hepatitis were negative, and an extensive panel of autoimmune hepatitis was also negative. Serum levels of carcinoembryonic antigen and carbohydrate antigen 19-9 had normal values.

Further investigations were conducted in order to establish the etiology of the compressing mass and the cause of left-sided portal hypertension.

Besides the compression noticed in the gastroscopy, the ultrasonography also showed a clearly outlined hypoechoic outer lesion in contact with the stomach (Figure 1). The lesion had a slightly irregular shape but clearly outlined margins, and it had a well-defined wall, without neo-vascularization or invasion in the adjacent structures. The peripheral interfaces were well maintained. In order to further validate our diagnosis, endoscopic ultrasound was chosen to examine the patient. Some of the differential diagnoses considered before EUS were pancreatic pseudocysts, gastric adenocarcinoma or lymphoma, excluded by the gastroscopy. Mucinous cystic tumors of the pancreas have similar features, and we also considered GISTs, neurogenic tumors, pancreatic heterotopia, neuroendocrine tumors, pancreatic pseudocysts and mesenteric cysts. However, due to the characteristics outlined through ultrasonography and eco-endoscopy, we were confident that the diagnosis was a GDC.

EUS established the diagnosis of a gastric duplication cyst, a homogenous lesion with regular margins, emerging from the submucosal layer, in contact with the greater curvature of the stomach, compressing the antrum and pylorus and measuring 102/80/60 mm. As a specific feature of duplication cysts, there is a concentric contraction of the cystic wall, a “ring contraction”, pathognomonic for this kind of malformation. EUS also showed that, due to the large diameter of the duplication cyst, it also compressed the splenic vein, which caused portal vein dilatation and the occurrence of left-sided portal hypertension. The spleen was orthotopic, without structural modifications, and it presented normal dimensions. Due to the suspicion of a duplication cyst, fine-needle aspiration was considered, but, due to the portal hypertension, esophageal varices and isolated gastric varices, it was not performed in order to prevent bleeding.

**Figure 5:**
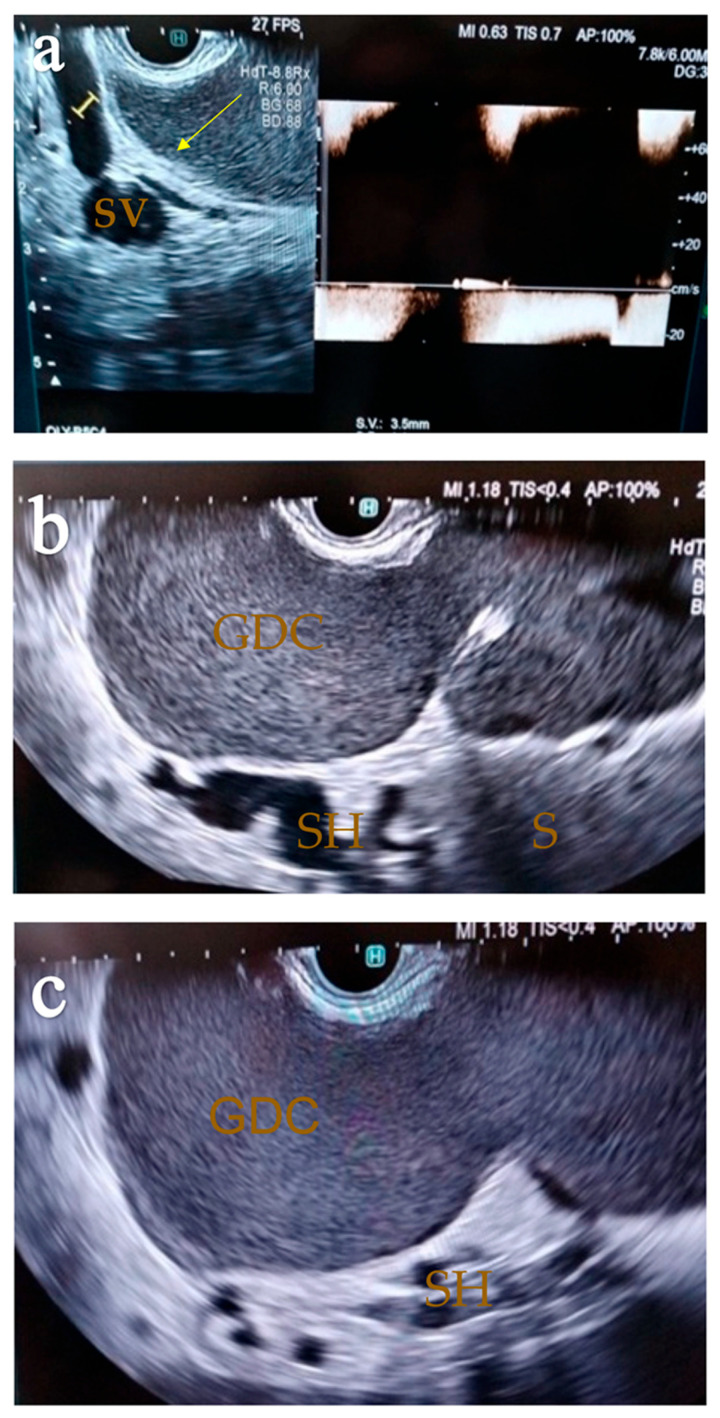
EUS view: (**a**) compression of the cyst on the splenic vein; SV—splenic vein. (**b**) GDC—gastric duplication cyst; SH—splenic hilum; S—spleen. (**c**) GDC—gastric duplication cyst with splenic hilum dilatations; SH—splenic hilum. (Personal collection).

Due to the fact that a GDC preoperative diagnosis is challenging and almost 65% of patients are misdiagnosed, the patient was also examined using computer tomography (CT), which supported our diagnosis. The CT scan was conducted as a preoperative assessment, and it showed a clearly delineated peri-gastric hypoattenuating polylobate lesion adjacent to the great curvature of the stomach, with a thin wall, homogeneous content with densities of 22–24 HU, with the contrast outlet only at the parietal level and dimensions of approx. 102/80/60 mm. The lesion was in contact with the visceral side of the spleen/splenic hilum, the tail of the pancreas and the left renal vessels. Other collaterals’ distribution of the veins was not described by the radiologists in the CT scan. The lesion had a slightly irregular shape but clearly outlined margins. The wall was well encapsulated and well defined (Figure 5).

Although the mass compressed the splenic hilum, it did not invade the vessels, acting as a benign tumoral mass. The risks posed by the cyst consisted of the fact that it could undergo erosion, ulceration, volvulus or malignant transformation. The cyst described was identified as non-communicating, which could result in mucosal necrosis due to increased fluid compression. Consequently, this could lead to bleeding within the cyst or perforation into the peritoneal cavity.

**Figure 6:**
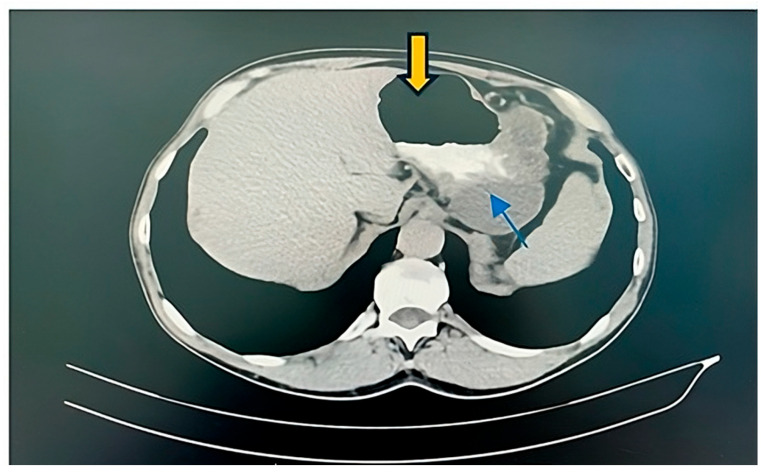
CT image: A clearly outlined peri-gastric hypoattenuating polylobate lesion (blue arrows) is seen adjacent to the great curvature of the stomach (yellow arrow), with a thin wall, homogeneous content and the contrast outlet only at the parietal level; its dimensions are approx. 102/80/60 mm. (Personal collection).

**Figure 7:**
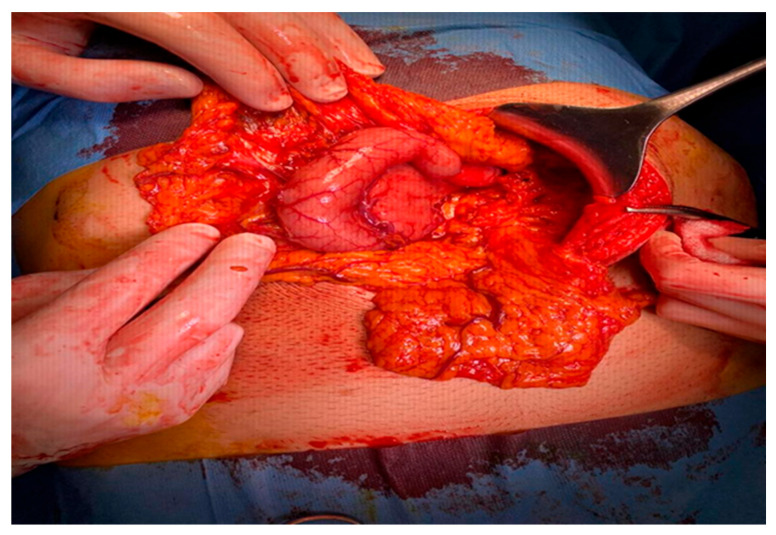
Gastric duplication cyst before resection in the operating room (Personal collection).

In the presented case, left-sided portal hypertension multiplied the risks, and surgical resection was considered the proper option to improve the patient’s quality of life due to persistent abdominal pain, the compression of other organs and the development of portal hypertension (Figure 5, Figure 6 and Figure 7).

### 2.1. Surgery

The patient underwent surgery, considering the complete resection of the GDC. The large compressive mass occupied the greater curvature to the pylorus and, due to the compression of the pylorus, gastric evacuation was difficult, so the patient presented gastric stasis. Laparoscopic intervention was attempted, but due to marked adhesion syndrome, classical surgery was considered the best option.

The cystic masses were resected, and non-communicating cysts were identified, occupying the entire greater curvature and exerting compression on the pylorus, as well as the splenic vein, resulting in portal hypertension. The patient was discharged without complications. At the seven-day follow-up, the patient continued to show no post-surgery disturbances, and digestion was not affected by the cyst resection. Additionally, the gastric stasis was resolved, and the abdominal pain and transit distress were remitted.

At the six-month follow-up, abdominal and digestive symptoms were remitted according to the anamnestic and clinical evaluation.

**Figure 8:**
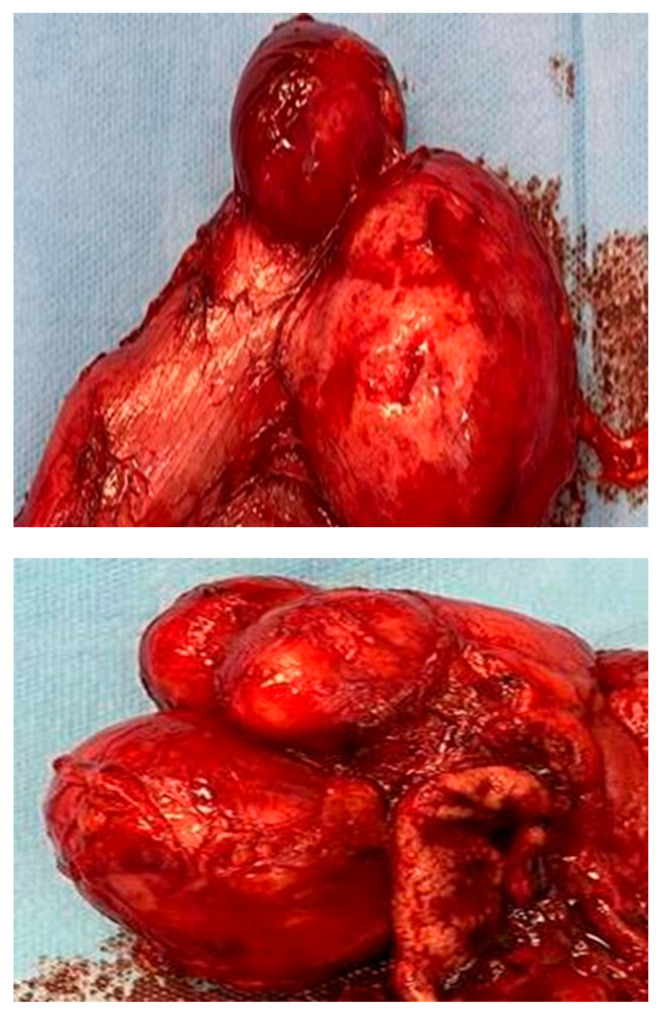
Gastric duplication cyst: two retrogastric tubular formations in contact with the greater gastric curvature without communication between them or with the gastric cavity, with the distal one extending up to the level of the pylorus, communicating with the proximal tubular formation at the level of the great curvature (Personal collection).

During the resection process, the following observations were made: multiple soft tumors with a smooth surface resembling the serosa of the stomach wall and two retrogastric tubular formations in contact with the greater gastric curvature, without communication between them or with the gastric cavity. The distal one extended up to the level of the pylorus and was found in the retrogastric space at the level of the omental bursa (Figure 8).

### 2.2. Histopathological Result

**Figure 9:**
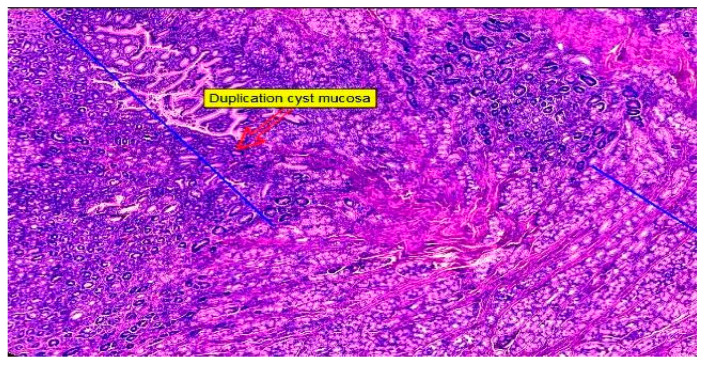
Hematoxylin-eosin staining 20×: gastric mucosa on the right in close contact with duplication cyst wall on the left (Personal collection).

The histopathological evaluation revealed a hyperplastic gastric mucosa, with prominent gastric crypts and abundant gastric mucous-secreting glands. The greater curvature of the stomach wall displayed a longitudinal dissection beginning in the submucosa, undoubtedly passing through the muscularis propria and invariably ending in the sub serosa, forming a well-defined dilatation, a multicavity cyst. The cyst was also lined by a hyperplastic gastric mucosa formed by a simple columnar epithelium, with prominent villi suggesting an intestinal metaplasia at this level and well-defined gastric mucous-secreting glands (Figure 9).

Toward the end, the lining of the space was reduced in thickness, as well as the foveolar lining, and it was replaced by a small strip of large cells with irregular borders, abundant ground-glass eosinophilic cytoplasms and small round hyperchromatic nuclei. Following this critical examination, the diagnosis of a gastric duplication cyst was made, with a biphasic mucosal lining: at the proximal end, there was an intestinal-type thick layer with villi and an abundance of glandular elements; at the distal end, the mucosal layer was thinner, being composed of parietal and mucous-secreting cells with a chaotic arrangement and a loss of the surface epithelium. No cellular atypia or dysplasia was found upon conducting the classic hematoxylin–eosin examination. The small blood vessels that feed the gastric and cystic walls were congested, but they had intact walls with a normal histological appearance and permeable lumen.

## 3. Discussion

**Figure 10:**
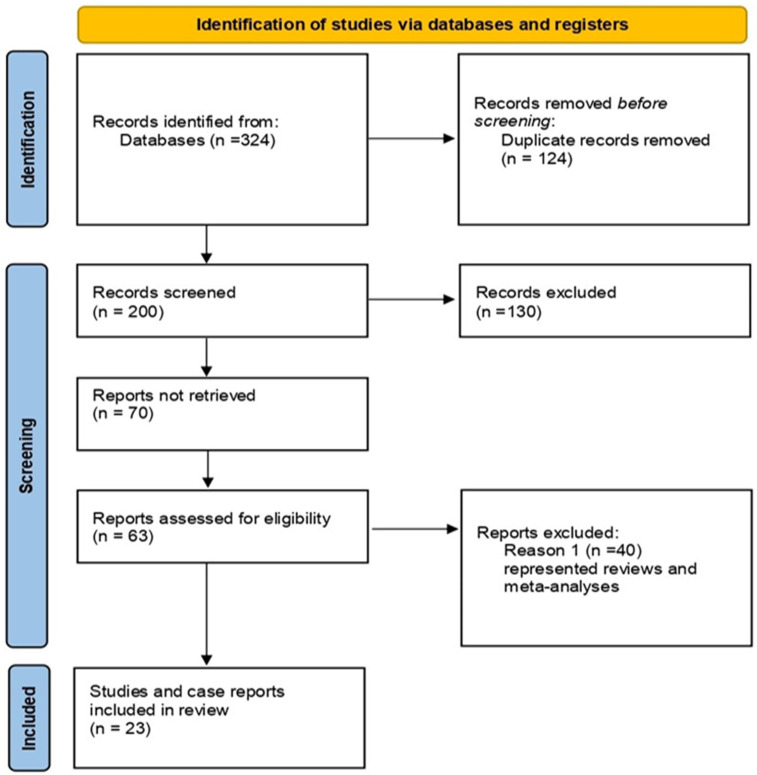
Research strategy. The inclusion criteria were original reports and reviews describing ultrasonography in gastric duplication cysts or malformations. The literature search yielded 324 studies, reviews and meta-analyses in total, via manual and electronic searching, including 124 duplicates, and another 200 articles were selected for screening. A total of 130 of these were excluded because they described other types of duplication cysts and, in the end, 70 articles were evaluated for eligibility. After the final search, a total of 63 articles were considered in the final analysis, with 23 of these representing original reports/case reports and studies; 40 were reviews and meta-analyses, which were excluded. The search, conducted up to December 2023, covered English-language publications in databases such as Web of Science, PubMed, Excerpta Medica Database, Google Scholar and UptoDate. Search terms used across these databases included variations and combinations of keywords related to gastric duplication cysts.

The pathology of gastric duplication cysts remains a controversial and relatively understudied subject in the literature, primarily due to the rarity of cases. There are ongoing debates regarding the presentation modalities of this pathology, especially from an imaging perspective, the sequence of investigations and how to enhance the efficiency of diagnosing this duplication cyst. To seek answers, we conducted a review of the specialized literature and noted the scarcity of cases concerning gastric duplication cysts in adult patients. Figure 10 summarizes the research strategy according to PRISMA (*The Preferred Reporting Items for Systematic Reviews and Meta-Analysis guidelines 2020*) criteria.

As observed in the case described in this paper, the symptoms of gastric duplication cysts are often nonspecific, and the diagnosis heavily relies on imaging studies. Symptoms commonly found in patients with this condition include diffuse abdominal pain, nausea, vomiting, dyspepsia, weight loss, dysphagia, with changes in the physical examination such as abdominal tenderness and potentially palpable epigastric mass. Despite the diverse range of symptoms associated with this condition, it is noteworthy that the early signs which led to the identification of the gastric duplication cyst in our patient are common across most case reports. Thus, we highlighted symptoms such as abdominal pain, weight loss and vomiting [10,18,19,20,21,22,23,24,25]. Although unlikely, this symptomatology should raise a red flag for practitioners regarding a possible gastric duplication cyst.

Advancements in ultrasonography and echo-endoscopy have significantly facilitated the diagnosis of rare cases of intestinal duplication. However, due to the infrequent occurrence of gastric duplication cysts in adults, there are currently no large-scale studies or meta-analyses providing indisputable evidence regarding the diagnostic and therapeutic protocols. Complex statistical analyses have not been performed to address questions regarding incidence, complications or predisposing factors. Nevertheless, there are several interesting cases presented in the literature, which have been summarized in Table 1, including the imaging diagnostic method and therapeutic action. The cases exhibit considerable diversity, with implications on surrounding organs varying based on the dimensions of the cystic formation. These factors complicate the diagnosis and may explain why only a small percentage (approximately one-third of cases) are correctly identified. The therapeutic approach must also adapt to the extent of the cyst and its impact on other organs.

In our case, EUS has shown superiority in diagnosis compared to CT. There is a higher risk of confusion with the latter method, and the challenge arises from the difficulty in discerning between cystic formations with varying contents. On the other hand, this does not seem to be the case for F-FDG-PET/CT [26]. Mohamad Abdalkader et al. presented the case of a 28-year-old adult who reported persistent abdominal pain and nausea [18]. EUS showed a gastric duplication cyst wall with a multi-layered aspect and an echogenic component in the mass. Endoscopic ultrasound displayed high accuracy in diagnosing GDC, but the patient postponed the surgery so, after one year, the symptoms persisted, and surgical cyst resection was the best option. Bhatia et al. presented another case involving a transonic cyst with multiple layers closely adjacent to the distal gastric body and antrum. The diagnosis of a gastric duplication cyst was established, with exclusion of GIST or malignancy, based on the detailed description provided by endoscopic ultrasound [27]. Efficient exclusion of malignancy was also achieved by Seijo et al., who described a gastric duplication cyst based on endoscopic ultrasound characteristics. They highlighted an anechoic cyst adjacent to the muscularis propria, which was continuous with the gastric wall and exhibited benign features [28]. It should be noted that Seijo advocates for fine-needle aspiration (FNA) for definitive diagnosis (understandably, given the potential for misdiagnosis) and exclusion of malignancy. However, each physician should carefully weigh the decision, as the procedure carries certain risks that cannot be overlooked and may lead to significant complications [29,30]. Thus, we can affirm that, in line with other studies, EUS represents the gold standard for making the diagnosis (excluding malignancy), determining the dimensions of the GDC and noting other potential complications.

In 1990, only a few instances of malignant transformation of gastric duplication cysts were documented in the literature [31]. However, in more recent cases, such as the one described by author Mohamed Abdulla et al., there have been instances of malignant transformation into adenocarcinoma. Although recent statistics on large patient cohorts confirming the exact rate of cyst transformation are lacking, we still emphasize the potential for malignant transformation in gastric duplication cysts and highlight the importance of surgery in preventing further complications [19].

The treatment of GDCs remains debatable, as there are multiple methods that can be followed depending on the size of the cyst and the patient’s symptoms. While some authors advocate for conservative treatment, others promote surgery as a safe method to reduce the risk of malignancy [1,32,33]. Jai P. Singh et al. described two cases: the first was diagnosed using CT and exploratory laparotomy, while the second was diagnosed through EUS, which provided higher accuracy in diagnosis. EUS revealed a cystic lesion measuring 3.5 × 2.5 cm, continuous along the gastric wall and surrounding the muscularis propria, suggestive of duplication cysts. For both cases, surgical management was considered the optimal choice due to the presenting symptoms and the potential risks of complications [10]. Similarly, for the case we presented, particularly due to portal hypertension, surgery was the primary recommendation, and the results were promising.

**Table 1:** The main articles presenting cases of gastric duplication cysts.

Authors	Sex/Age	Symptoms	Location/Size (cm)	CT	US	EUS	Preoperative Diagnosis	Surgery
[18]	F/28	Persistent abdominal pain and nausea	Greater curvature of the stomach	NA	Cystic mass divided in two lobules located in the right upper quadrant, having smooth internal surface with dependent echogenic component	Multilayered aspect of the gastric duplication cyst.	GDC	Laparoscopic excision of the cyst (1 year later)
[10]	M/27	Back pain, loss of appetite	Adheres to posterior wall of stomach near to the greater curvature	Nonseptated cystic mass, in relation to pancreatic tail	NA	NA	GDC	Excision of cystic mass with resection of adjacent stomach
	F/28	Epigastric pain, vomiting	Medial wall of antrum and pylorus	4 cystic lesions	NA	Multiple intramural cystic lesions	GDC	Antrectomy and truncal vagotomy
[20]	M/25	Abdominal pain, epigastric fullness, vomiting, dysphagia, dyspepsia, anemia.	Posterior wall of the stomach	Well-marginated cystic lesion.	NA	NA	GIST	Cyst resected laparoscopically
[19]	M/51	2 day history of melena	Greater curvature of the stomach	Cystic mass that has a septum dividing it into 2 parts	NA	NA	GDC Adenocarcinoma	Total gastrectomy
[21]	F/8	Abdominal pain and vomiting.	Near the left lobe of the liver and stomach cavity	Cystic mass occupying the epigastric and left hypochondrial area	Upper abdominal cystic mass, which was 12 × 8 × 2.5 cm in size and was located between the left lobe of the liver and stomach cavity	NA	Liver cyst	Cyst resected
[34]	F/52	No	Between the stomach and body of the pancreas	Low density mass without intensification in the arterial phase and portal phase		Anechoic cystic lesion located close to the body of the pancreas	Pancreatic mucinous cystic neoplasm	Laparoscopic resection of the cyst
[22]	F/44	Abdominal pain, nausea and constipation	Between stomach and body of the pancreas	Cystic lesion in the middle of stomach and body of the pancreas	NA	Normal pancreatic echotexture and a cyst free of internal septations or associated masses but compressed the stomach	Pancreatic mucinos cyst	En bloc resection of the cyst with a portion of the posterior wall of the stomach
[23]	M/67	Anorexia, epigastric pain and severe weight loss	Between the gastric fornix and the body of the pancreas	A cystic lesion located between the gastric fornix and the body of the pancreas.	NO	The cyst, which originated from the demonstrated early diffuse enhancement following the injection of SonoVue. FNA was performed	GDC	Total gastrectomy Histology confirmed the presence of a gastric duplication cyst and malignant degeneration
[12]	F/34	Renal colic	Pancreatic tail	Cystic lesion of the tail of the	Cystic image at the right renal lobe	Cystic dense-walled formation in the tail of the pancreas FNA was performed	GDC	NA

CT: computed tomography; US: ultrasonography, EUS: endoscopic ultrasonography; FNA: fine needle aspiration, M: male; F: female; GDC: Gastric duplication cyst; NA: not available.

Important aspects of our case presentation are the existence of esophageal varices and left-sided portal hypertension as a result of long-time compression on the splenic vein. In our review of the current literature, we did not encounter a case presentation describing compression on the splenic vein, left-sided portal hypertension, or pyloric obstruction due to the large dimensions of the cyst. The limitation of our case presentation is that we could not carry out angio-CT to evaluate other potential collaterals. Comparable to other studies, the histopathological findings revealed no dysplastic cells, which excluded malignancy and improved the curative prognosis of the patient. In pediatric patients, due to the fact that cysts are discovered earlier, the chances of complications, transformation into adenocarcinoma or the development of portal hypertension are lower. This rare case of left-sided portal hypertension due to compression of the large duplication cyst may be seen as a warning for pediatric practitioners regarding the possible complications of benign duplication cysts, and it highlights the need to remove cysts using endoscopic methods (if the dimensions allow it) or surgery [35,36].

It should be noted that due to the presence of gastric atrophy, several possible factors that could have caused this change need to be considered. Among the most important is the increasing pressure in the cavity resulting from secreting mucosa or the possibility of a minor communication of the gastric duplication cyst with the gastric cavity, which could not be identified during the investigations conducted.

The study’s limitations are attributed to the absence of similar cases within the same hospital, thereby preventing the feasibility of executing a comprehensive statistical analysis.

## 4. Conclusions

Gastric duplication cysts, rare congenital anomalies in adults, may present with symptoms like abdominal pain or bleeding. Our case is unique in the literature as the only instance where a GDC determined left-sided portal hypertension, gastric stasis and pylorus obstruction.

The case report and literature review underscore the crucial role of EUS and abdominal ultrasound in accurately diagnosing gastric duplication cysts. It is significant to emphasize that the challenge of distinguishing between a gastric duplication cyst and gastric adenocarcinoma, or even possible pancreatic mucinous cysts, in preoperative imaging, may complicate achieving an accurate diagnosis. The treatment remains controversial and must be carefully chosen, but surgical intervention offers significant advantages by drastically reducing the risk of malignancy and symptoms in large-sized cysts, while also contributing to achieving a precise diagnosis.

## Data Availability

The original contributions presented in the study are included in the article, further inquiries can be directed to the corresponding author.
